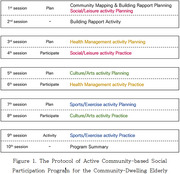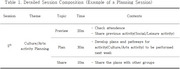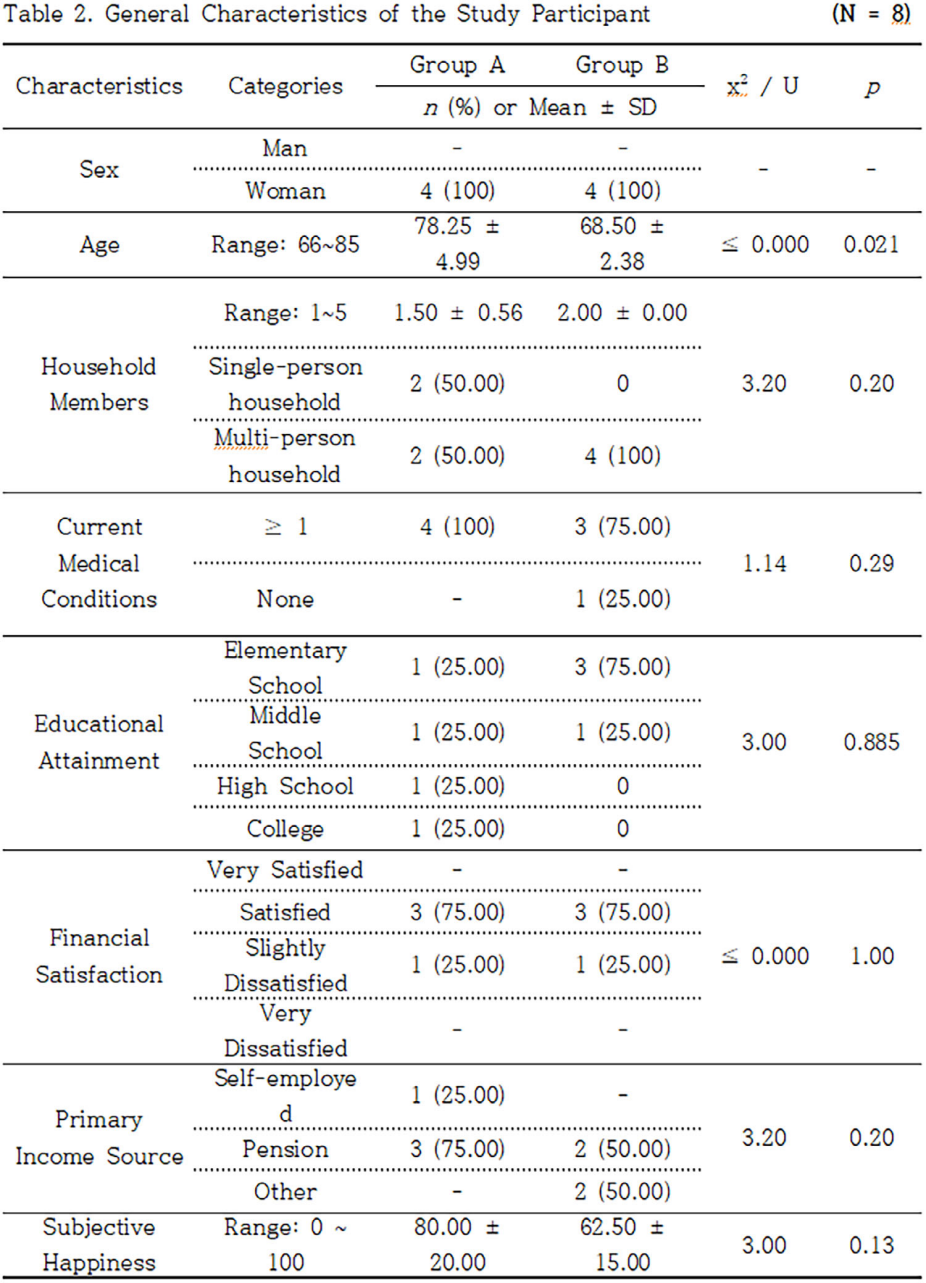# A Pilot Study on the Effectiveness of Community‐Driven Social Participation Programs for Older Adults’ Well‐Being

**DOI:** 10.1002/alz70860_101534

**Published:** 2025-12-23

**Authors:** Jiwon Shin, MuWon Lee, Hyun Yang, ChaeYoung Lee, Hae Yean Park

**Affiliations:** ^1^ Graduate School, Yonsei University, Wonju, Wonju, Korea, Republic of (South); ^2^ Yonsei University, Wonju‐si, Gangwondo, Korea, Republic of (South); ^3^ College of Software and Digital Healthcare Convergence, Yonsei University, Wonju, Heungup‐meon, Korea, Republic of (South)

## Abstract

**Background:**

Social prescribing has been widely implemented in the United Kingdom; however, it remains underdeveloped in South Korea. As the country enters a super‐aged society, there is an urgent need for effective interventions to address social isolation and enhance well‐being among older adults. This study aims to evaluate the effectiveness of an Active Community‐Based Social Participation (ACoSP) program in reducing social isolation and improving the well‐being of community‐dwelling older adults in South Korea.

**Method:**

A pilot study was conducted over a one‐month period with eight older adults residing in Wonju, South Korea. Participants engaged in a total of ten intervention sessions, consisting of alternating planning and implementation sessions. The intervention incorporated a community asset mapping approach and a structured participation framework targeting cultural, physical, and social activities. Outcome measures included the Social Isolation and Social Network (SISN) scale, the Yonsei Lifestyle Profile‐BREF (YLP‐BREF), the General Self‐Efficacy Scale, and a satisfaction survey. Wilcoxon signed‐rank tests were conducted to assess pre‐post differences.

**Result:**

The intervention group showed an increase in physical activity and social participation scores; however, the changes were not statistically significant. Qualitative interviews revealed improvements in self‐efficacy, increased community participation, and strengthened social networks. Participants reported enhanced motivation to engage in community activities and shared positive experiences regarding program support and interactions with peers. Satisfaction survey results indicated a high perceived need for the program.

**Conclusion:**

The findings suggest that an Active Community‐Based Social Participation program holds promise in addressing social isolation and improving well‐being among older adults in South Korea. Further research with a larger sample and extended intervention duration is needed to validate the effectiveness and sustainability of such programs within the community healthcare framework.